# Pulse Wave Velocity Measurements by Magnetic Resonance Imaging in Neonates and Adolescents: Methodological Aspects and Their Clinical Implications

**DOI:** 10.1007/s00246-022-02894-0

**Published:** 2022-04-09

**Authors:** Simon Lundström, Jonas Liefke, Einar Heiberg, Erik Hedström

**Affiliations:** 1 Clinical Physiology, Department of Clinical Sciences Lund, Lund University, Skåne University Hospital, Lund, Sweden; 2 Wallenberg Centre for Molecular Medicine, Lund University, Lund, Sweden; 3 Diagnostic Radiology, Department of Clinical Sciences Lund, Lund University, Skåne University Hospital, Lund, Sweden

**Keywords:** CMR, Neonates, Adolescents, Pulse wave velocity, Standardization

## Abstract

Pulse wave velocity (PWV) by cardiovascular magnetic resonance (CMR) lacks standardization. The aim of this study was to investigate methodological aspects of PWV measurements by CMR in neonates and adolescents. A computer phantom was created to validate the temporal resolution required for accurate PWV. Fifteen neonates and 71 adolescents underwent CMR with reference standard 3D angiography and phase-contrast flow acquisitions, and in a subset coronal overview images. Velocity and flow curves, transit time methods (time-to-foot (TTF), maximum upslope, and time-to-peak (TTP)), and baseline correction methods (no correction, automatic and manual) were investigated. In neonates, required timeframes per cardiac cycle for accurate PWV was 42 for the aortic arch and 41 for the thoracic aorta. In adolescents, corresponding values were 39 and 32. Aortic length differences by overview images and 3D angiography in adolescents were − 16–18 mm (aortic arch) and − 25–30 mm (thoracic aorta). Agreement in PWV between automatic and manual baseline correction was − 0.2 ± 0.3 m/s in neonates and 0.0 ± 0.1 m/s in adolescents. Velocity and flow-derived PWV measurements did not differ in either group (all *p* > 0.08). In neonates, transit time methods did not differ (all *p* > 0.19) but in adolescents PWV was higher for TTF (3.8 ± 0.5 m/s) and maximum upslope (3.7 ± 0.6 m/s) compared to TTP (2.7 ± 1.0 m/s; *p* < 0.0001). This study is a step toward standardization of PWV in neonates and adolescents using CMR. It provides required temporal resolution for phase-contrast flow acquisitions for typical heartrates in neonates and adolescents, and supports 3D angiography and time-to-foot with automatic baseline correction for accurate PWV measurements.

## Background

Arterial stiffness is a predictor for long-term cardiovascular disease [1]. Pulse wave velocity (PWV) is an established surrogate marker for arterial stiffness and an independent predictor of cardiovascular mortality and morbidity [1, 2]. Whereas carotid-femoral applanation tonometry is commonly used for measuring PWV, this method lacks in precision. Cardiovascular magnetic resonance (CMR) imaging agrees with invasive measurements of PWV but is limited by lack of standardization [3–9]. More specifically**,** required temporal resolution, flow plane locations, use of baseline correction, and applied PWV curve analysis methods differ between studies [1, 6–10]. Differences in PWV values may thus be due to both physiological and methodological differences, which limits comparison between studies and clinical use of reference values.

Dorniak et al. [6] showed that the required temporal resolution for PWV measurements in adults is 35 timeframes per cardiac cycle, corresponding to a temporal resolution of 30 ms. Required temporal resolution for children is however unknown. Additionally, it is unclear whether standard CMR coronal overview images acquired as part of a routine CMR examination are sufficient in resolution for measuring aortic length or whether the more accurate reference standard for vessel length assessment, 3D angiography, is required. The lack of standardization limits clinical applicability and particularly complicates comparison of data between centers.

The overall aim of this study was therefore to investigate methodological aspects of PWV measurements by CMR, as a means toward standardization in neonates and adolescents. Specifically, the aims were to investigate (1) the required temporal resolution of phase-contrast flow data for accurate PWV measurements for different aortic lengths using a computer phantom; (2) impact on PWV using standard coronal overview images and non-contrast-enhanced 3D angiography for centerline vessel length; (3) velocity-based and flow-based curves for PWV; (4) impact on PWV using different transit time methods; and (5) impact of baseline correction on PWV.

## Materials and Methods

### Study Population and Protocol

Data from 15 prospectively included neonates and 71 adolescents who underwent cardiovascular magnetic resonance imaging at Skåne University Hospital between 2014 and 2020 were used. An additional adolescent was included for computer phantom construction and was not further incorporated in analyses. The regional Ethics Review Board (Lund, Sweden) approved the study. All participants, and their guardians when appropriate, provided written informed consent before participation in the study.

### Computer Phantom

The pulse wave velocity computer phantom methodology from Dorniak et al. [6] was used to establish the required temporal resolution needed for accurate PWV measurements. Phantom vessel lengths were set to approximately the minimum vessel lengths of the current populations, i.e., 25 mm for neonates’ aortic arch, 60 mm for neonates’ thoracic aorta and adolescents’ aortic arch, and 150 mm for adolescents’ thoracic aorta. Post-acquisition analysis of the study population revealed sufficient timeframes per cardiac cycle for construction of a high-resolution computer phantom for neonates, but not for adolescents. This was due to a limitation in reconstructed number of timeframes. As a result, one additional adolescent (17-year-old female, 162 cm, 55 kg) was included and used for the phantom construction only. Computer phantoms with a reference pulse wave velocity between 2 and 10 m/s were generated to cover the normal pulse wave velocity interval for neonates and adolescents [11], while also including large margins for potential confounders. A representative high-resolution ascending flow curve from each group was upsampled to 10,000 timeframes per cardiac cycle. A simulated corresponding descending flow curve was created by shifting the ascending flow curve in time with a delay computed as the vessel length (aortic arch or thoracic aorta) divided by the reference PWV (i.e., 2 m/s, 4 m/s, 6 m/s, 8 m/s, 10 m/s). Thereafter the descending flow curve was scaled in amplitude with 60% to mimic the blood diversion to the aortic arch branches and finally smoothed with a Gaussian smoothing filter with a width of 2,000 timeframes. The flow profile was then downsampled to 20–60 timeframes per cardiac cycle to mimic image acquisition with different temporal resolutions. For each combination of reference PWV and timeframes per cardiac cycle, the PWV was calculated using the TTF method on the downsampled ascending and descending flow curves and compared to the reference PWV. Differences were presented as % error. The full code for the phantom can be found as part of the open source code distribution for the software Segment.

### Pulse Wave Velocity by CMR in Neonates and Adolescents

Participants were imaged in supine position using a 1.5 T MR scanner (Philips Achieva, Best, the Netherlands, or Siemens Aera, Erlangen, Germany). For adolescents a 32-channel phased-array cardiac coil (Philips) or 18-channel and spine coil combination (Siemens) was used according to clinical routine. For neonates a small flexible coil was chosen. Further, neonates were positioned in a vacuum infant immobilizer and either imaged using feed-and-sleep or if this was not possible, using chloral hydrate 25 mg/kg administered rectally. A 2D phase-contrast gradient recalled echo sequence with retrospective ECG gating was used for quantitative flow measurements. For both neonates and adolescents, two phase-contrast flow planes were acquired; one positioned in the ascending aorta as part of the clinical standard protocol which also includes the descending aorta immediately after the aortic arch, albeit potentially angulated to the vessel, and one at the level of the diaphragm. For aortic length measurements in adolescents, 3D oblique sagittal slices covering the thoracic aorta (non-contrast enhanced 3D angiography) and coronal slices covering the entire thorax (coronal overview) were acquired. A subset of adolescents (n = 49) had both 3D non-contrast-enhanced angiography and thoracic coronal overview images acquired for comparison of aortic length measurements. The coronal overview images were based on a balanced SSFP sequence with inplane resolution 1.66 mm and slice thickness 8 mm and slice gap 2.64 mm, whereas the 3D angiography was based on a clinical routine T2-prepared balanced steady-state free precession sequence with isotropic resolution 0.88 mm (Philips) or 0.55 mm (Siemens). In neonates a clinical routine 3D black-blood T1-weighed non-contrast-enhanced angiography sequence was used with isotropic resolution 1.04 mm (Siemens). Typical image parameters for the quantitative flow phase-contrast sequence were TR/TE 4.92/2.67 ms, flip angle 20°, 1.5 × 1.5 × 5 mm^3^ (Siemens) for neonates. Corresponding parameters for adolescents were TR/TE 9.15/5.54 ms, flip angle 15°,1.2 × 1.2 × 6 mm^3^ (Philips); and TR/TE 4.92/2.67 ms, flip angle 20°, 1.5 × 1.5 × 5 mm^3^ (Siemens). Reconstruction was set to a maximum of 50 timeframes per cardiac cycle for neonates and 35 for adolescents. In the one adolescent scanned for construction of the computer phantom, flow data were collected using the Siemens scan protocol but with the reconstruction limit set at 70 timeframes per cardiac cycle to avoid assessment errors. Velocity encoding was 150–250 cm/s to optimize velocity resolution and avoid aliasing. Temporal resolution (ms) was calculated as heartrate duration divided by timeframes per cardiac cycle.

Image analysis was performed using the software Segment version 3.2 R9405 (http://segment.heiberg.se, Medviso AB, Lund, Sweden) [12]. Three observers assessed velocity and volumetric flow curves in the ascending aorta, descending aorta after the aortic arch and at the level of the diaphragm, and length measurements in the aortic arch and thoracic aorta. The observers had 2 years’ (observer 1, assessed coronal overview data; SL), 5 years’ (observer 2, assessed 3D angiography data and flow data; JL), and 21 years’ (confirmed data from observer 1 and observer 2; EHed) CMR experience. A subset of 15 data sets (5 neonates and 10 adolescents) were randomly chosen and delineated by observers 1 and 2 for interobserver assessment. Aortic arch and thoracic aorta lengths by 3D angiography were used for length comparison between observers and the PWV method with least bias and variability was used for transit time comparisons.

**Fig. 1:**
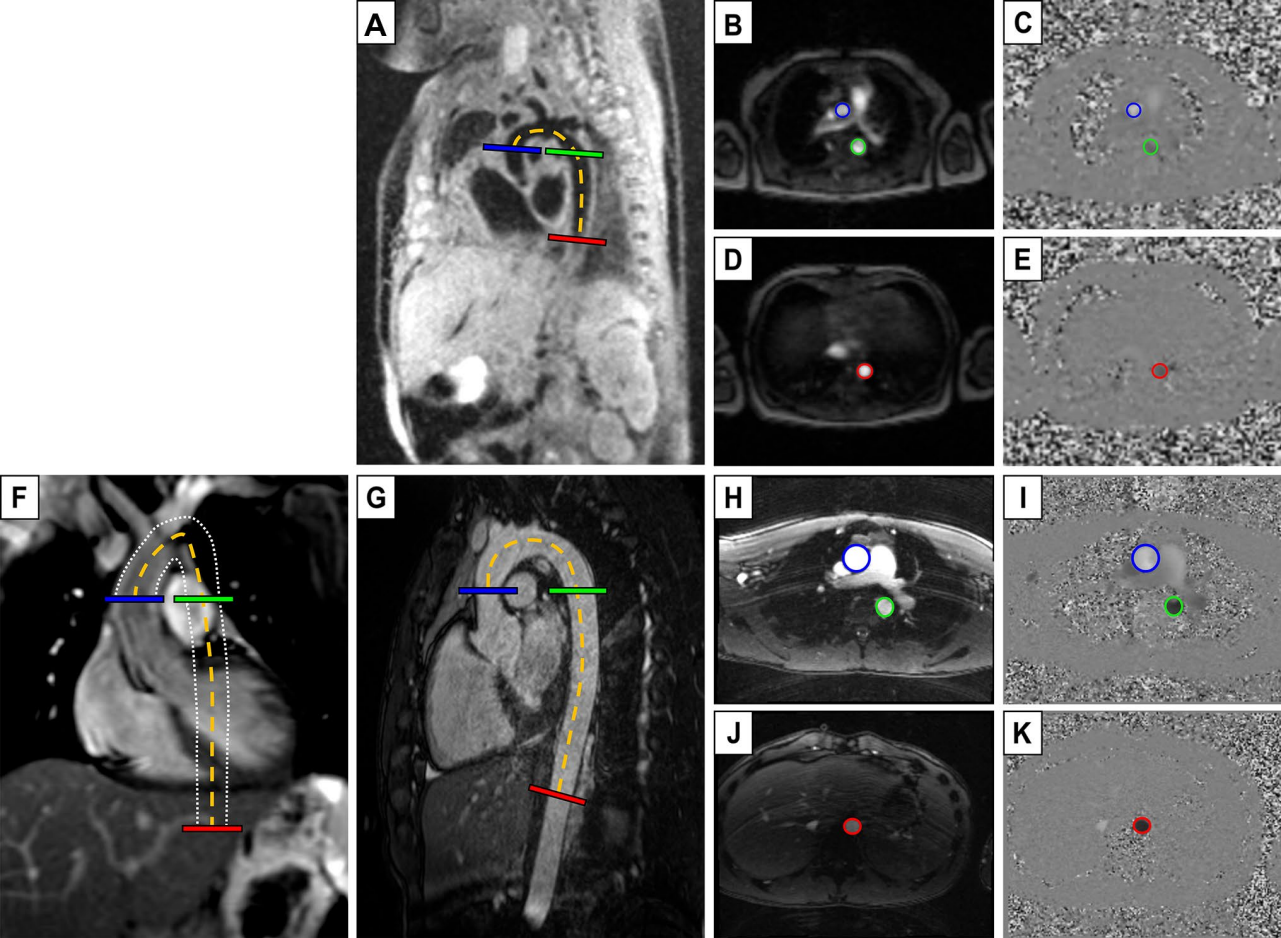
Image delineations for aortic length and flow measurements. Neonatal black-blood (**A**) and adolescent white-blood (**G**) 3D angiography, and adolescent thoracic coronal overview image with white dotted lines outlining the aortic walls throughout the 2D image stack (**F**), all with length delineation (Δd) (dashed orange) and perpendicular flow imaging planes in the ascending aorta (blue), descending proximal aorta (green) and descending aorta at the level of the diaphragm (red). Magnitude and quantitative phase-contrast images at ascending and proximal descending aorta (**B–C**, **H–I**) and descending aorta at the level of the diaphragm (**D–E**, **J–K**), corresponding to the flow imaging planes localizations. Worth noting is that both the ascending and descending flow plane in the aortic arch (**B–C**, **H–I**) are collected in a single flow plane as in clinical routine which may result in angular error in the descending aorta, whereas the flow plane at the level of the diaphragm is perpendicular

Vessel length (Δd), i.e., blood traveling distance between flow planes, was measured in the reference standard 3D angiography images and in 2D coronal overview images. For both image types a manual frame-by-frame centerline vessel tracking method was used (Fig. 1). Lengths reported are from the reference standard 3D angiography unless otherwise specified.

**Fig. 2:**
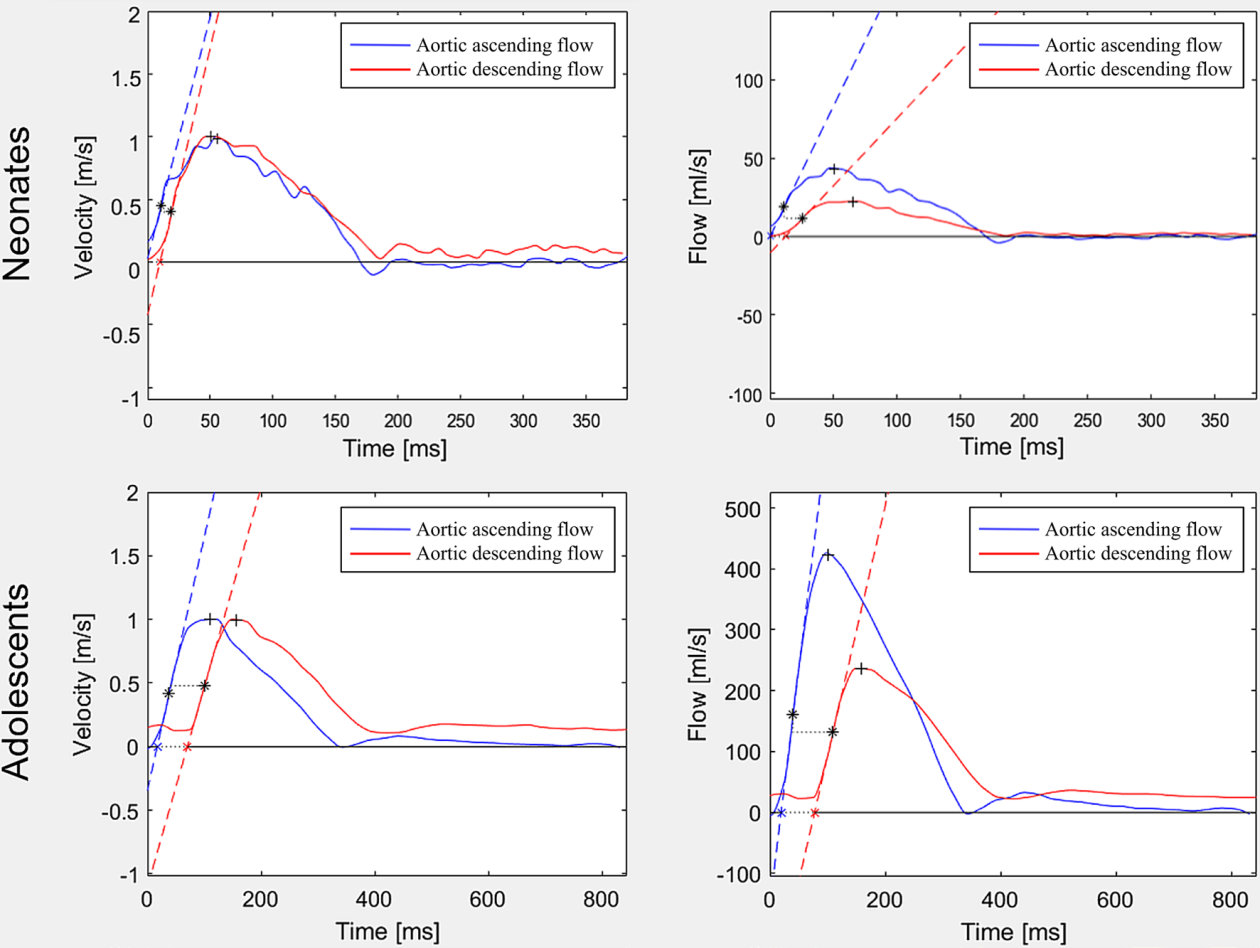
Velocity and flow curves. Curves for neonates (top row) and adolescents (bottom row) based on velocity (left column) and flow (right column), all without baseline correction. The difference in curve shape may be caused by redirected blood volume to the carotids and subclavian arteries. This leads to the decreased magnitude of the aortic descending flow curve (right), while the velocity curve is more constant in shape. The difference in curve shape had least impact on the time-to-foot transit method, which may indicate this method’s intrinsic robustness (c.f. Figure 9). Also, note the challenge to assess PWV in neonates using the TTP method as the descending curve peak time-point may end up prior to the ascending curve peak time-point (top left), resulting in negative PWV values. * = time points used for maximum upslope PWV calculation, +  = time points used for TTP PWV calculation (c.f. Figure 3 for details on transit time methods)

Velocity and flow curves were assessed using automatic segmentation with manual correction where needed [12] with vessel circumference delineated throughout the cardiac cycle in magnitude images supported by phase-contrast images as appropriate and analyzed according to Dorniak et al. [6] for PWV (Fig. 2).

**Fig. 3:**
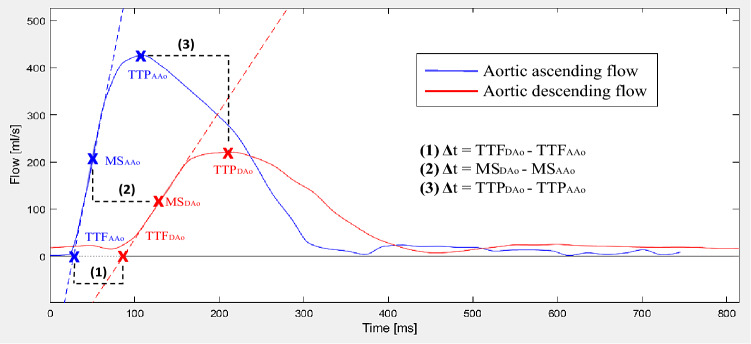
Flow curve analysis. Example of analysis methods demonstrated on a flow-based pulse wave velocity (PWV) curve. Note that all algorithms also apply to velocity-based curves (not shown). Blue line corresponds to flow in the ascending aorta (AAo) and red line to flow in descending aorta at the level of the diaphragm (DAo). Pulse wave velocity was calculated by aortic length divided by transit time (Δd/Δt). Transit time was estimated using three different algorithms. Algorithms and corresponding points on the flow curves are shown for time-to-foot (TTF), maximum upslope (MS), and time-to-peak (TTP). The algorithms were based on the baseline intersection of an automatically generated tangent superimposed at the point of maximum upslope (TTF); the maximum upslope incline (maximum upslope); and the maximum curve magnitude (TTP)

**Fig. 4:**
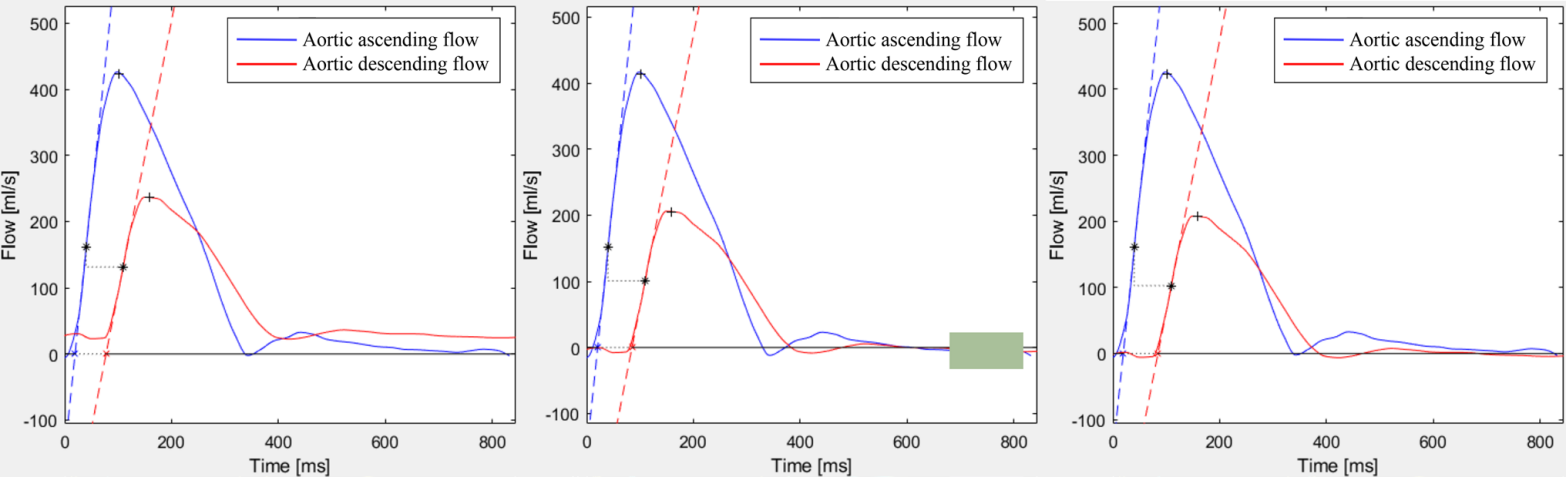
Baseline correction methods. Pulse wave velocity curves with no (left), automatic (middle), and manual baseline correction (right). Automatic correction uses the average from the 80th–95th % segment of the flow curve to approximate the ascending and descending curve, marked with a green box. For manual baseline correction, emphasis was instead put on leveling the curves prior to the upslope. Baseline correction was only applicable for the time-to-foot transit method, as it is the only method that relates to the baseline

Three transit time (Δt) methods were compared as shown in Fig. 3; namely (1) time-to-foot (TTF), (2) maximum upslope, and (3) time-to-peak (TTP). The algorithms were based on the baseline intersection of an automatically generated tangent superimposed at the point of maximum upslope (TTF); the maximum upslope incline (maximum upslope), and the maximum curve magnitude (TTP). For TTF, which is baseline dependent, baseline correction was applied both automatically and manually. For automatic baseline correction, a modified version of the algorithm previously described by Dorniak et al. [6] was applied, using instead the mean of the 80th–95th % segment of the flow curves (Fig. 4). For manual correction, this study instead proposed to use the segment prior to the systolic upslope, to avoid errors related to the diastolic phase of the cardiac cycle such as backward flow related to insufficiency. Gaussian smoothing was performed on all curves to optimize signal-to-noise ratio with sigma = 0.025. Pulse wave velocity was calculated as PWV = traveling distance (Δd) / transit time (Δt).

### Statistical Analyses

Statistical analyses were performed in GraphPad 9.2.0 for Windows (GraphPad Software, La Jolla, California, USA). Group characteristics and vessel lengths were not normally distributed and presented as median (range), whereas PWV comparisons were normally distributed and therefore presented as mean ± SD. Bland–Altman plots assessed agreement between methods with data presented as bias ± SD in text and bias and 95% limits of agreement (LoA; i.e., ± 1.96 SD) in graphs. Intra-individual variations between coronal overview images and the reference standard 3D angiography are presented as median and 2.5th–97.5th percentile. Interobserver variabilities are presented as mean ± SD. Paired t-test or repeated-measures ANOVA with Tukey correction post hoc assessed group differences. P values < 0.05 were considered to indicate statistically significant differences.

## Results

### Computer Phantom

**Fig. 5:**
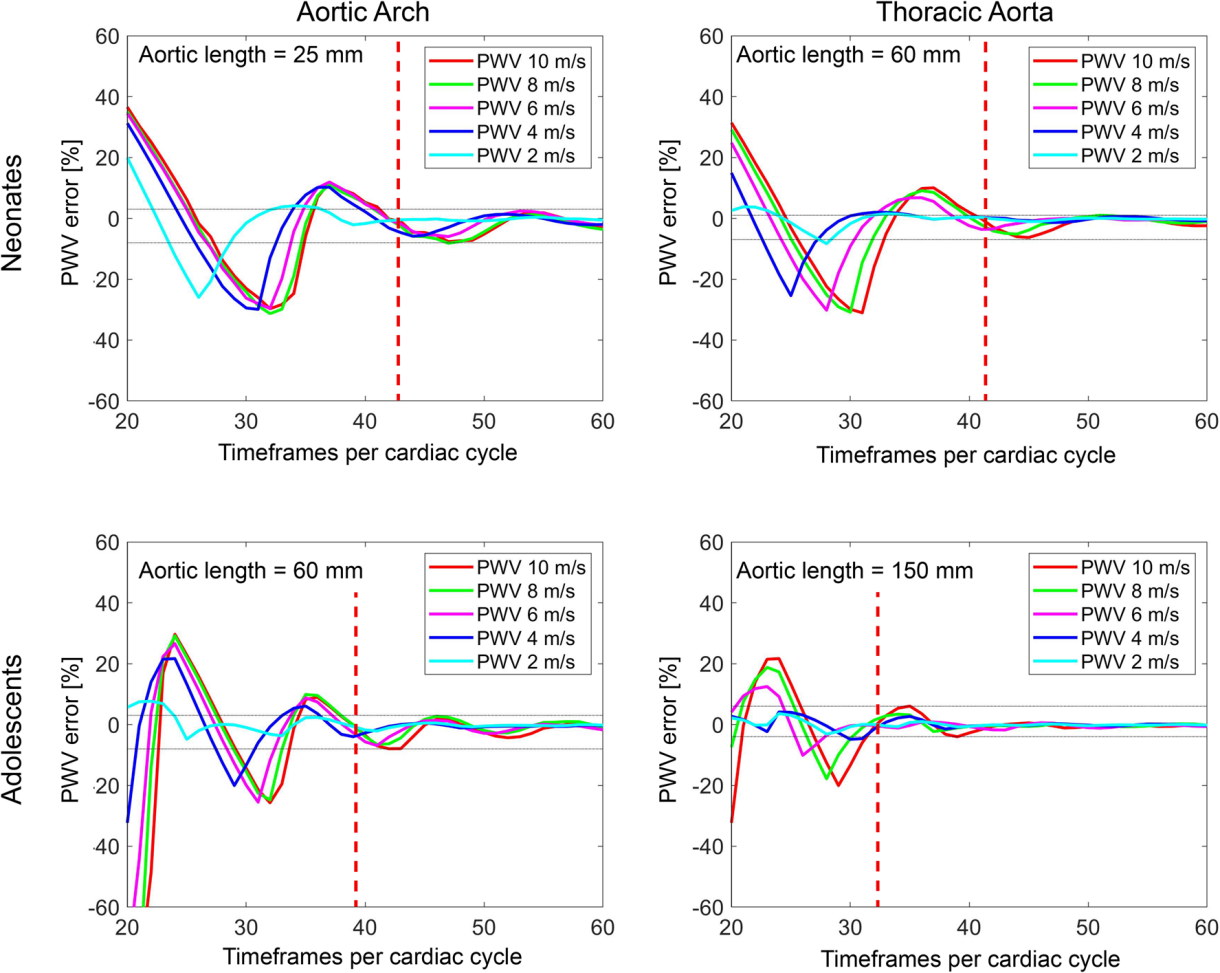
Minimum timeframes per cardiac cycle for neonates and adolescents. Computer phantom based on neonatal (top row) and adolescent (bottom row) flow data for aortic lengths 25 mm (top left, i.e., neonatal aortic arch), 60 mm (top right i.e., neonatal thoracic aorta, and bottom left, i.e., adolescent aortic arch), and 150 mm (bottom right, i.e., adolescent thoracic aorta) and for different pulse wave velocities. The aortic lengths presented above approximates the shortest lengths in the current population. Vertical red dashed lines denote the cut-off for required timeframes per cardiac cycle for all pulse wave velocities. Horizontal gray dotted lines denote the mean error (min–max) after the cut-off. For both shorter aortic lengths and higher pulse wave velocities, the required timeframes per cardiac cycle for accurate pulse wave velocity measurements and the pulse wave velocity error at inadequate temporal resolutions increased. X-axis offset between neonatal and adolescent curves is likely related to the shorter diastolic period in neonates due to their higher heartrate

Figure 5 shows the computer phantom results as timeframes per cardiac cycle, independent of heart rate. For a neonatal flow curve profile and an aortic length > 25 mm (i.e., shortest neonatal aortic arch), 42 timeframes per cardiac cycle resulted in a PWV error range of − 8–2%, and for an aortic length > 60 mm (i.e., shortest neonatal thoracic aorta) 41 timeframes per cardiac cycle resulted in a PWV error range of − 6–1%. Corresponding number of timeframes for an adolescent flow profile and aortic length > 60 mm (i.e., shortest adolescent aortic arch) and for an aortic length > 150 mm (i.e., shortest adolescent thoracic aorta) were 39 timeframes per cardiac cycle (PWV error range − 8–3%) and 32 timeframes per cardiac cycle (PWV error range − 4–6%), respectively. Fewer timeframes per cardiac cycle and particularly so combined with higher PWV and shorter pulse wave traveling distance yielded larger errors, with neonatal aortic arch showing the largest PWV error of approximately 30% at 33 timeframes per cardiac cycle and a PWV of 8 m/s.

### Pulse Wave Velocity by CMR in Neonates and Adolescents

Table 1 shows group characteristics for neonates and adolescents. Reconstructed timeframes per cardiac cycle was 44 in neonates, corresponding to a temporal resolution of 10 ms for an average heart rate of 138 bpm, and 35 in adolescents corresponding to a temporal resolution of 22 ms for an average heart rate of 75 bpm.

**Table 1:** Group characteristics for neonates and adolescents

	Neonates	Adolescents
Participants (females)	15 (47%)	71 (51%)
Age (neonates: days; adolescents: years)	14 (6–45)	14 (13–17)
Weight (kg)	3.3 (2.8–6.5)	56 (37–90)
Height (cm)	50 (45–58)	165 (148–189)
Aortic arch length (mm)	30 (23–59)	86 (62–124)
Thoracic aortic length (mm)	68 (55–83)	188 (149–258)
Heart rate (beats per minute)	138 (108–160)	75 (44–128)

Absolute values (%) and median (range)

### Aortic Lengths

**Fig. 6:**
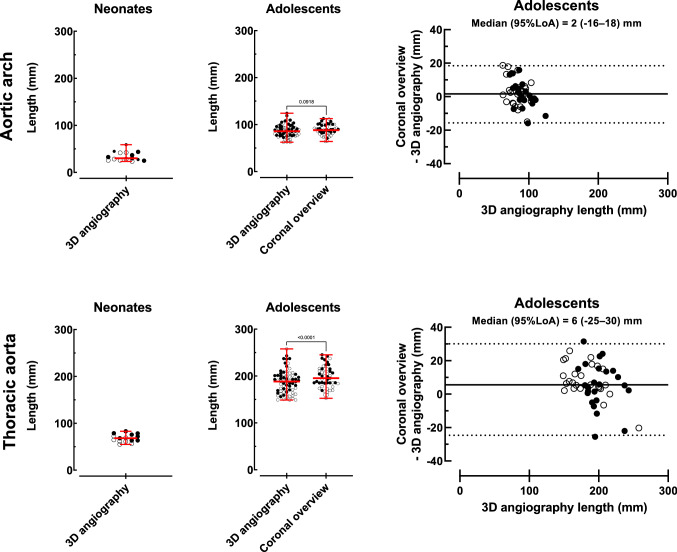
Aortic length measurements. Aortic arch (top row) and thoracic aorta (bottom row) length measurements in neonates (*n* = 15, left column), adolescents (*n* = 71, middle column), and intra-individual variation between adolescent coronal overview images and the reference method 3D angiography (*n* = 49, right column). Solid circles represent males and open circles females. Red lines with error bars denote median (range). Solid black lines represent bias and dashed lines represent 95% limits of agreement

Figure 6 shows vessel length measurements and agreement. In neonates the median aortic centerline length by 3D angiography was 30 (23–59) mm for the aortic arch and 68 (55–83) mm for the thoracic aorta. In adolescents, the median aortic arch length was 86 (62–124) mm by the reference standard 3D angiography and 88 (64–113) mm by overview images (*p* = 0.09), and median thoracic aortic length was 188 (149–258) mm by the reference standard and 195 (153–245) mm by overview images (*p* < 0.0001). Variations between the reference standard 3D angiography and coronal overview images were − 16–18 mm for aortic arch length and − 25–30 mm for thoracic aortic length. Interobserver variability for 3D angiography length measurements for neonates and adolescents combined were – 2 ± 2 mm.

### Impact of Baseline Correction on PWV

**Fig. 7:**
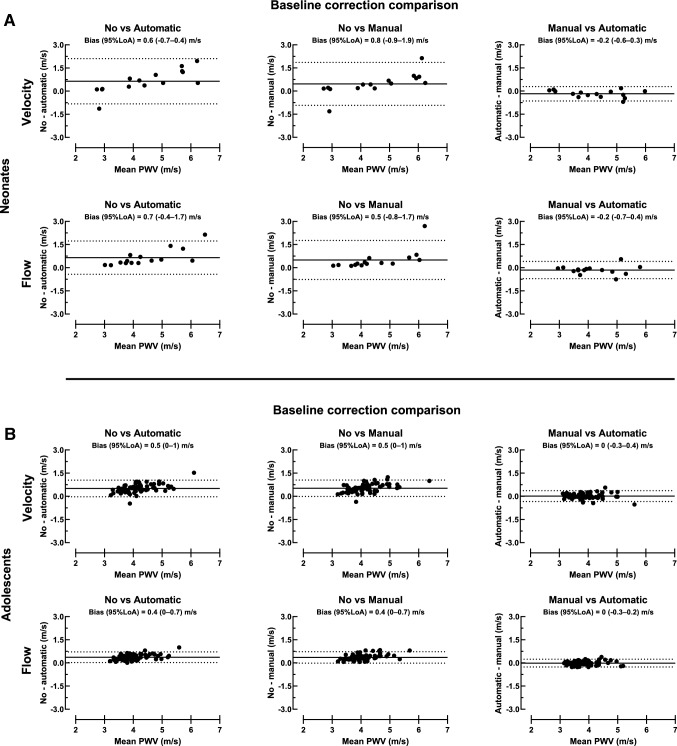
Baseline correction comparison. Bland Altman plots for neonates (**A**; top panel) and adolescents (**B**; bottom panel) with comparison of baseline correction for thoracic aorta velocity (top row) and flow curves (bottom row). Agreement between no and automatic baseline correction (left), no and manual baseline correction (middle), and manual and automatic baseline correction (right) is presented in both rows. Manual and automatic baseline correction agreed for both velocity and flow curves, which indicates that these baseline correction methods can be used interchangeably in neonates and adolescents. Solid black lines represent bias and dashed lines represent 95% limits of agreement

Figure 7A shows agreement between baseline correction methods for thoracic aorta PWV in neonates. Pulse wave velocity bias between automatic and manual baseline correction was – 0.2 ± 0.2 m/s for velocity curves and − 0.2 ± 0.3 m/s for flow curves. Corresponding bias was 0.5 ± 0.7 m/s and 0.5 ± 0.6 m/s for manual and no baseline correction and 0.6 ± 0.7 m/s and 0.7 ± 0.5 m/s for automatic and no baseline correction.

Figure 7B shows agreement between baseline correction methods for thoracic aorta PWV in adolescents. Pulse wave velocity bias between automatic and manual baseline correction was 0.0 ± 0.2 m/s for velocity curves and 0.0 ± 0.1 m/s for flow curves. Corresponding bias was 0.5 ± 0.3 m/s and 0.4 ± 0.2 m/s for manual and no baseline correction and 0.5 ± 0.3 m/s and 0.4 ± 0.2 m/s for automatic and no baseline correction.

Table 2 shows thoracic aorta PWV differences related to baseline correction methods. In neonates, velocity-based PWV by TTF with no baseline correction was higher (4.8 ± 1.6 m/s) than with automatic baseline correction (4.2 ± 1.0 m/s) (*p* = 0.01). In adolescents, PWV was higher using both velocity and flow curves for TTF with no baseline correction (4.4 ± 0.6 m/s and 4.2 ± 0.5 m/s) compared with automatic (3.9 ± 0.5 m/s and 3.8 ± 0.5 m/s) and manual baseline correction (3.9 ± 0.5 m/s and 3.8 ± 0.4 m/s; all *p* < 0.0001). Further, velocity-based PWV by TTF with no baseline correction was higher (4.4 ± 0.6 m/s) than the corresponding flow-based PWV (4.2 ± 0.5 m/s) (*p* < 0.001).

**Table 2:** Pulse wave velocity (m/s) differences related to baseline correction methods

	Velocity	Flow	*p* value
Neonates			
No baseline correction	4.8 ± 1.6	4.8 ± 1.3	> 0.99
Automatic baseline correction	4.2 ± 1.0*	4.1 ± 0.9	> 0.99
Manual baseline correction	4.3 ± 1.1	4.3 ± 0.9	> 0.99
Adolescents			
No baseline correction	4.4 ± 0.6	4.2 ± 0.5	< 0.0001
Automatic baseline correction	3.9 ± 0.5***	3.8 ± 0.5***	0.07
Manual baseline correction	3.9 ± 0.5***	3.8 ± 0.4***	0.76

All values are based on thoracic aorta measurements and the time-to-foot algorithm. Mean ± SD

*p < 0.05 compared to no baseline correction

*** *p* < 0.0001 compared to no baseline correction

### Impact of Transit Time Methods on PWV

**Fig. 8:**
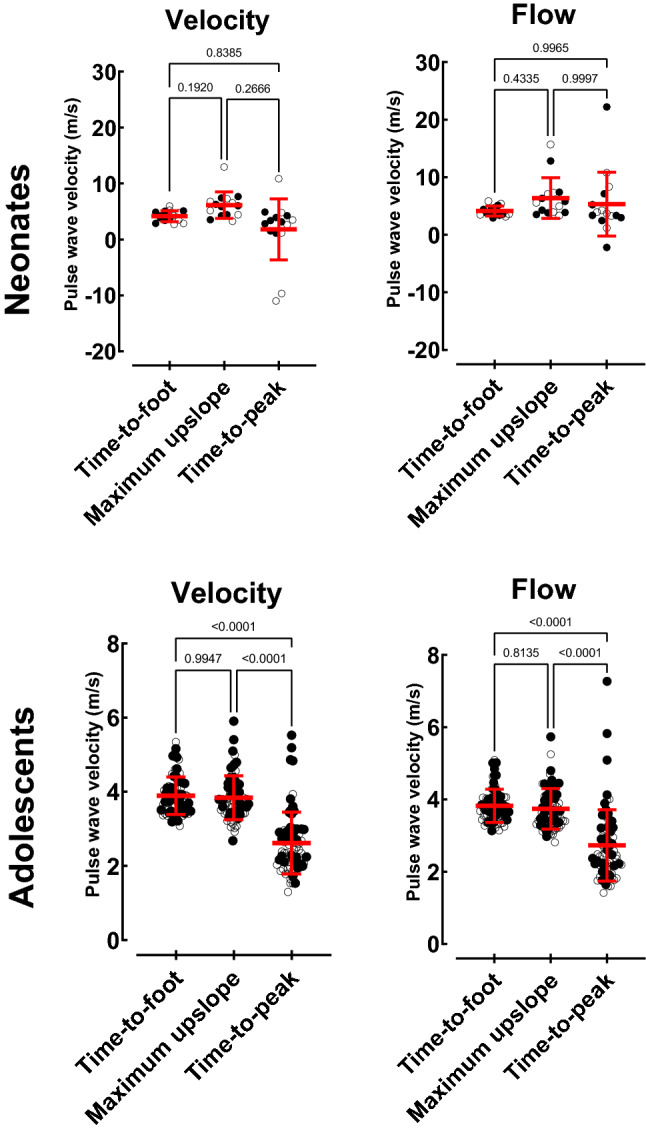
Transit time measurements. Method comparisons for neonates (top row) and adolescents (bottom row) with thoracic aorta PWV for velocity (left) and flow (right) derived time-to-foot with automatic baseline correction, maximum upslope, and time-to-peak algorithms. Time-to-peak was lower for both velocity and flow curves. Negative PWV values are due to curve shapes with no definitive peak, leading to errors in the TTP algorithm (c.f. Fig 2). Note that the TTP algorithm therefore cannot be recommended in neonates and adolescents. Solid circles represent males and open circles females. Red line with error bars denotes mean ± SD

Figure 8 shows transit time method comparisons. In neonates, thoracic aorta PWV based on velocity curves were 4.2 ± 1.0 m/s for TTF, 6.1 ± 2.4 m/s for maximum upslope, and 1.8 ± 5.4 m/s for TTP. Corresponding PWV based on flow curves were 4.1 ± 0.9 m/s, 6.4 ± 3.5 m/s, and 5.3 ± 5.6 m/s.

In adolescents, thoracic aorta PWV based on velocity curves were 3.9 ± 0.5 m/s for TTF, 3.8 ± 0.6 m/s for maximum upslope, and 2.6 ± 0.8 m/s for TTP. Corresponding PWV based on flow curves were 3.8 ± 0.5 m/s, 3.7 ± 0.6 m/s, and 2.7 ± 1.0 m/s. Time-to-peak thus yielded lower PWV than maximum upslope (*p* < 0.0001) and TTF (*p* < 0.0001) for both velocity and flow curves in adolescents.

**Fig. 9:**
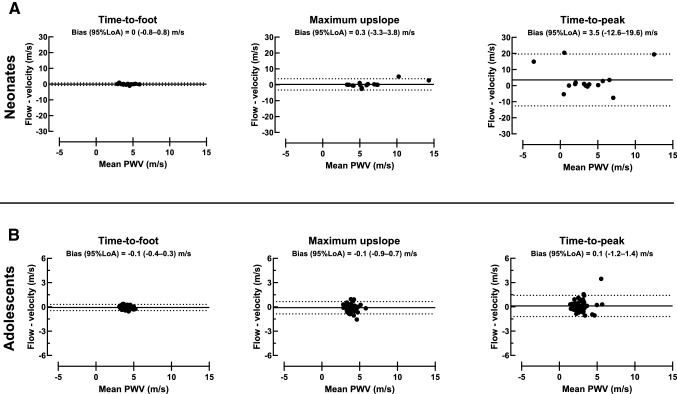
Transit time agreement. Bland–Altman plots for neonates (top row) and adolescents (bottom row) with comparison of thoracic aorta velocity and flow-based maximum upslope (left), time-to-foot with automatic baseline correction (middle), and time-to-peak (right) algorithms. For both neonates and adolescents, limits of agreement were narrower for time-to-foot, which may be an indicator of the transit time estimator’s intrinsic robustness. Note the difference in y-axis ranges for neonates and adolescents. Negative PWV values are due to curve shapes with no definitive peak, which worked poorly with the TTP algorithm (c.f. Fig 2). Solid black lines represent bias and dashed lines represent 95% limits of agreement

Figure 9 shows agreement between thoracic aorta velocity and flow curves for each transit time method. Time-to-foot yielded PWV 0.0 ± 0.4 m/s for neonates and − 0.1 ± 0.2 m/s for adolescents. Corresponding values were for maximum upslope 0.3 ± 1.8 m/s and − 0.1 ± 0.4 m/s, and for TTP 3.5 ± 8.2 m/s and 0.1 ± 0.7 m/s.

### Pulse Wave Velocities

**Fig. 10:**
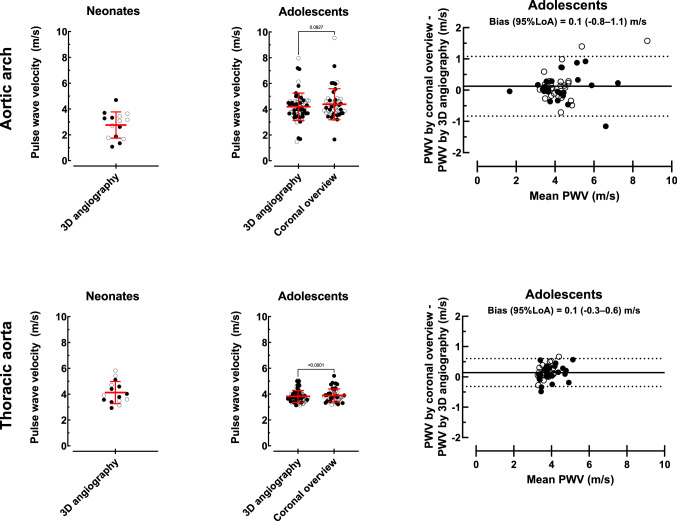
Aortic pulse wave velocity measurements. Aortic arch (top row) and thoracic aorta (bottom row) flow-based time-to-foot PWV with automatic baseline correction measurements in neonates (*n* = 15, left column) and adolescents (*n *= 71, middle column) and intra-individual differences between adolescent coronal overview images and the reference method 3D angiography (*n* = 49, right column). Solid circles represent males and open circles females. Red lines with error bars denote mean ± SD. Solid black lines represent bias and dashed lines represent 95% limits of agreement

Figure 10 shows neonatal and adolescent PWV based on TTF with automatic baseline correction. Neonatal PWV based on centerline distance by 3D angiography was 2.8 ± 1.0 m/s for the aortic arch and 4.1 ± 0.9 m/s for the thoracic aorta. Adolescents’ aortic arch PWV was 4.3 ± 1.1 m/s based on centerline distance by 3D angiography and 4.4 ± 1.2 m/s when centerline distance was based on coronal overview images (*p* = 0.08). Corresponding values for adolescents’ thoracic aorta PWV were 3.8 ± 0.4 m/s and 3.9 ± 0.5 m/s (*p* < 0.0001). Variation in adolescents’ PWV derived from coronal overview and 3D angiography length measurements was more prominent (− 0.8–1.1 m/s) for the aortic arch than for the thoracic aorta (− 0.3–0.6 m/s). Interobserver variability for flow PWV by TTF with automatic baseline correction was 0.0 ± 0.1 m/s, corresponding to an error of − 1 ± 2%.

## Discussion

This study showed how methodological aspects influence PWV values by CMR in neonates and adolescents. The differences in measured PWV values confirm the hypothesis that measurement methods cannot be used interchangeably. This in turn limits comparability between existing studies, clinical applicability of previously presented reference values, and potentially the ability to use retrospective data due to too few timeframes per cardiac cycle and low spatial resolution in images for vessel centerline measurement. This study thereby serves as a mean toward standardization of PWV measurements by CMR.

More specifically, this study showed that: (1) the required timeframes per cardiac cycle for minimum PWV errors was 42 for the aortic arch and 41 for the thoracic aorta in neonates and 39 and 32 for corresponding vessel segments in adolescents; (2) standard coronal overview images cannot replace 3D angiography without risk of intra-individual measurement errors; (3) PWV based on velocity or flow curves does not differ in a young population; (4) time-to-foot with automatic baseline correction has high agreement and low variability and should be used in favor of maximum upslope, whereas time-to-peak cannot be recommended; and 5) automatic baseline correction agrees with manual baseline correction and can be used in subjects without retrograde diastolic flow.

### Computer Phantom

This study showed that both shorter aortic lengths as related to age [13] or flow plane acquisition location, and increased PWV as related to age, grade of hypertension, or other cardiovascular diseases [14] risks yielding larger errors at inadequate temporal resolution in both neonates and adolescents. All these factors are thus important to consider in order to ensure sufficient temporal resolution in the individual patient. Pulse wave velocity of the aortic arch can be derived from a single flow plane covering both the ascending aorta and descending aorta immediately after the aortic arch, but requires increased temporal resolution to compensate for the short distance between flow planes. The applicability of low and high temporal resolution acquisitions have been investigated [15, 16] but only Dorniak et al. [6] has of yet dissected the impact of a continuum of timeframes per cardiac cycle and temporal resolutions, despite being addressed as an important aspect already in 2014 [1]. This study presented temporal resolution as the heartrate independent measurement timeframes per cardiac cycle. For comparison between studies and for standardization of clinical application an increased transparency regarding acquired timeframes per cardiac cycle is beneficial.

### Pulse Wave Velocity by CMR in Neonates and Adolescents

The current study tested the hypothesis that standard coronal overview images could be used interchangeably with the reference method non-contrast enhanced 3D angiography for measuring accurate blood traveling distance and to calculate PWV in the aortic arch and thoracic aorta. On a group level, a small and likely clinically insignificant difference between 3D angiography and coronal overview was observed. However, when comparing the intra-individual length and corresponding PWV variability for 3D angiography and coronal overview, the variation is both large and unpredictable (Figs. 6 and 10). Differences in slice thickness, slice gap, and number of slices covering the aorta likely explain differences in measured aortic lengths and correspondingly PWV. Therefore, as non-contrast-enhanced 3D angiography can be acquired in only a few minutes it is preferred for accurate PWV calculations also in the young and healthy. The same logic can be applied for the aortic arch where, due to its shorter length and its curvature, accurate centerline length measurements are even more critical. This was also shown in the current study with larger PWV variation in the aortic arch. Retrospective analysis of clinical data sets with only coronal overview images and a single flow acquisition including both the ascending and descending aorta is therefore not recommended, but if used, should only be applied on a group level and with caution.

The current study showed no difference in PWV based on velocity or flow curves. Flow curves are commonly used for PWV by CMR but are affected by diversion of blood. For PWV measured in the aortic arch or thoracic aorta, blood mainly diverge into aortic arch vessels leading to a decline in blood volume of approximately 40% between the ascending and descending aortic flow measurement planes. This can be noted as the difference between the descending and ascending flow curves as shown in Fig. 2. Velocity curves are on the other hand routinely acquired by Doppler ultrasound for applanation tonometry-based PWV. They are less affected by diversion of blood but are instead susceptible to local variations in velocity [17]. Velocity curves are rarely used for PWV by CMR but may be advantageous in more challenging populations due to the more homogeneous curve shapes for transit time estimations. This however remains to be tested.

For baseline correction, automatic correction based on the 80th–95th % segment of the flow curve, i.e., late diastole, agreed with manual correction based on the more variable pre-systolic segment in both neonates and adolescents and for both velocity and flow curves, implying an adequate automatic algorithm. The previously proposed automatic method used the mean of the 62.5th–87.5th % segment of the flow curve [6], i.e., early diastole. Using the diastolic phase for correction may yield false results if extended to populations including patients with diastolic retrograde flow. By instead using a timepoint closer to the systolic upslope, as proposed in the current study, the influence of retrograde diastolic flow may be reduced. This hypothesis was not tested in the current healthy population, and a separate study including patients with retrograde diastolic flow is needed to confirm this hypothesis.

The commonly used transit time methods TTF, maximum upslope, and TTP [1, 6–10] were compared in the current study, showing that TTF with automatic baseline correction had the narrowest limits of agreement in both neonates and adolescents. This agrees with previous studies showing TTF to be a reproducible method also in other populations [1]. Importantly, neonatal PWV did not differ between methods, whereas in adolescents PWV by TTP was lower than when using TTF and maximum upslope. Time-to-peak not only underestimated PWV, but also risks reporting false negative flow as shown in Fig. 2. This also explains the considerably lower, albeit not statistically significant, PWV value for velocity-based TTP in neonates (1.8 ± 5.4 m/s) as compared to TTF (4.2 ± 1.0 m/s) and maximum upslope (6.1 ± 2.4 m/s). In addition, the current study assessed each method’s robustness, suggesting the use of TTF with automatic baseline correction as the method of choice in neonates and adolescents as it is more robust and less variable than the other tested methods. This will also increase comparability between hospitals and research studies, and use of reference values.

Reference values are available for aortic arch PWV by CMR in adolescents [9, 18]. In both these studies, a sufficient number of timeframes per cardiac cycle was used for flow assessment in the aortic arch, assuming their populations are comparable to the current population in terms of heart rate and aortic arch centerline distance. However, these previous studies used maximum upslope [9] and time-to-half peak [18] as transit time methods, which is important to consider when applying these reference values in relation to the method used locally, as it may lead to wrongly interpreted results.

Whether this suggested standardized method also is accurate compared to invasive measurements remains to be answered. Compared to the current methodological situation, however, the current study presents a reproducible and precise transit time method which is a step forward in increasing comparability and applicability both in clinics and research.

### Limitations

The current study did by design not include elderly and patients with cardiovascular disease and the results shown may not be directly transferable to these populations. It may be hypothesized that the differences shown between methods in the current young population are even larger in elderly and in patients with cardiovascular disease. Further, the current study had a reconstruction limit of 35 timeframes per cardiac cycle for flow data acquired in adolescents, which is slightly fewer than that shown to be needed in the adolescent aortic arch, but not thoracic aorta, by the computer phantom. Therefore, the presented adolescent aortic arch PWV values should be taken with caution. However, the method comparisons should not be affected as aortic arch PWV measurements was used only for comparison between use of 3D angiography versus coronal overview images for centerline distance.

## Conclusion

This study showed how methodological aspects influence PWV values by CMR in neonates and adolescents. It thereby serves as a mean toward standardization. Phase-contrast flow for assessment of PWV should be acquired with at least 42 and 41 timeframes per cardiac cycle in the neonatal aortic arch and thoracic aorta, respectively, and 39 and 32 timeframes per cardiac cycle for corresponding vessel segments in adolescents. This corresponds to temporal resolutions of 10 ms, 11 ms, 21 ms, and 26 ms, and can be applied to similar populations at their typical heartrates. Adequate number of timeframes and temporal resolution can be accomplished at the scanner by ensuring a sufficient reconstruction limit, which does not affect scan time. Further, 3D angiography should be used for vessel length measurements for accurate PWV, and the transit time method time-to-foot with automatic baseline correction is suggested.

## Data Availability

The datasets generated and/or analyzed during the current study are not publicly available due to sensitive information but are available in anonymized form from the corresponding author on reasonable request.

## Abbreviations

CMR: Cardiovascular magnetic resonance
PWV: Pulse wave velocity
TTF: Time-to-foot
TTP: Time-to-peak
